# Attitudes to aging, depression, and death anxiety among community-dwelling older adults: a cross-sectional correlational study

**DOI:** 10.1186/s12877-025-05839-3

**Published:** 2025-07-02

**Authors:** Hülya Kulakçı Altıntaş, Nilay Ercan Şahin

**Affiliations:** 1 Health Science Faculty, Nursing Department, Zonguldak Bülent Ecevit University, Esenköy, Kozlu, Zonguldak, 67600 Turkey; 2 Nursing Faculty, Hacettepe University, Adnan Saygun Stress D-Buildings 1. Floor, Samanpazarı, Ankara, 06100 Turkey

**Keywords:** Attitudes to ageing, Community-dwelling older adults, Death anxiety, Depression, Nursing

## Abstract

**Background:**

This study was conducted to evaluate the effects of attitudes to ageing and depression as predictors of death anxiety in community-dwelling older adults.

**Method:**

The study was a cross-sectional and correlational design and conducted with 400 participants who met the inclusion criteria and admitted to four different family health centers in Turkiye. Data were collected with the Individual Evaluation Form, Attitudes to Ageing Questionnaire, Geriatric Depression Scale and Templer Death Anxiety Scale.

**Results:**

According to the results, community-dwelling older adults have depressive symptoms and death anxiety; and the attitudes to ageing and depression can predict death anxiety among community-dwelling older adults.

**Conclusion:**

This result highlights that interventions should be planned in order to reduce death anxiety among community-dwelling older adults by taking the attitude to ageing and the symptoms of depression into account.

## Background

Ageing is a process of change that begins with the birth of a person and continues until death and it is associated with numerous and inevitable changes in physical, cognitive, and social functionality [1]. In the ageing process, an individual's attitude toward ageing becomes crucial in coping with the physical, mental, and social changes that occur [2]. Individuals develop an attitude towards ageing by being exposed to the culture of the society in which they have been grown up. If society has negative stereotypes against ageing, these negative stereotypes are internalized and affect the individual's physical, emotional, and cognitive processes when he/she gets older [3]. Physically, individuals may begin to perceive themselves as frail or incapable, leading to a decline in self-care behaviors and a lower level of activity [4]. Emotionally, the internalized ageism can contribute to feelings of inadequacy, depression, or anxiety, as the individual may feel they do not meet societal expectations of youth or vitality [5]. Cognitively, the belief in ageing as a time of inevitable decline may reduce the motivation to engage in stimulating activities or seek new learning opportunities, thereby accelerating cognitive decline [6]. Overall, when the individual’s experiences and behaviors align with society’s expectations, it perpetuates the cycle of ageing in a negative light [4]. Moreover, in this process, older adults' attitudes toward ageing lead to changes in the domains of psychosocial loss, physical change, and psychosocial growth. Negative emotions and experiences such as loneliness, depression, and social isolation are considered aspects of psychosocial loss. Perceptions related to changes in physical abilities, health status, and body image are classified under physical change. Meanwhile, awareness of positive changes in later life, such as wisdom, life experience, and personal growth, is regarded as psychosocial growth [7]. There are many studies evaluating the attitude towards ageing in different parts of society especially younger people [8–10]. However, the number of studies evaluating the attitude towards ageing among older adults individuals is limited [2, 11, 12]. Korkmaz Aslan et al. found that attitude to ageing is a factor that affects health-promoting behaviours among older adults [12]. In addition, it was also demonstrated that attitudes to ageing were associated with depressive symptoms among older adults [2, 11].

Studies have shown that depression is common in the older adults due to the changes experienced with ageing such as deterioration of health, decreased productivity and social isolation [13, 14]. Depression is a significant variable for older adults, and can be associated with impairments in physical and cognitive function as well as mortality [15]. One of the most prominent features which distinguish old age from other time periods is the proximity of the end of life [1]. This situation may cause older adults to experience death anxiety. Death anxiety is the inability to accept death as a part of life [16]. In fact, everyone may experience some level of death anxiety [17], but it is more pronounced among the older adults [18]. Woo and Bae found that depression was positively correlated with death anxiety. In particular, individuals with depression may experience deeper death anxiety because of their tendency to have more negative emotions [19]. Moreover, it has been stated that individuals with depression have higher death anxiety, and death anxiety is associated with various psychological variables such as depression, panic attacks and somatic symptoms [20]. Furthermore, depression is one of the most important factors influencing older adult's death anxiety [21].

As metioned above, attitudes toward ageing are deeply shaped by cultural values, traditions, and societal norms [22]. In collectivist cultures like Turkiye, ageing is often associated with respect, familial support, and intergenerational solidarity [23]. However, modernization, urbanization, and changing family structures have led to a shift in traditional perspectives on ageing, potentially increasing feelings of loneliness and social isolation among older adults [24]. These cultural transformations can also influence psychological well-being, as older individuals in Turkiye may experience heightened depression and death anxiety when their social roles and familial connections diminish [25, 26]. According to the literature, attitudes to ageing [27, 28], depression [29, 30] and death anxiety [31–33] are prevalent and disabling conditions for older adults that increase the risk of mortality and influence the quality of life negatively [34]. It is important to enhance the quality of the years that older adults live. The aim of nursing is to promote and protect the health of the individuals at every period of life. In line with this purpose, the goal of nursing care provided to older adults is to maintain an active and quality life [35]. In addition to meeting physical needs of older adults, nurses help them to cope with their emotional problems, to be self-efficient and to see themselves as valuable. The nurse plays an important role in determining the social support resources that will protect the mental health of the older adults in order to increase the quality of life, revealing the needs of them and planning and implementing the interventions [29]. In order to improve the quality of life and mental state of older adults, it is necessary to plan and implement nursing interventions by determining the attitudes to ageing, depression level and death anxiety among older adults. Therefore, this study was conducted to evaluate attitudes to ageing and depression as predictors of death anxiety in the community-dwelling older adults. Based on the results of this study, it is expected to shed a light on the interventions to be developed for the older adults and further research.

For this purpose, answers to the following questions were sought in this study:

1. What is the attitude towardageing of community-dwelling older adults?
2. What is the depression level of community-dwelling older adults?
3. What is the death anxiety level of community-dwelling older adults?
4. Are attitude to ageing and depression predictors of death anxiety among commumity-dwelling older adults?

## Methods

This study was conducted to evaluate the effects of attitudes to ageing and depression as predictors of death anxiety in community-dwelling older adults. This study was a cross-sectional and correlational design.

### Sample and participants

The population of the study consisted of 4,748 older adults aged 65 and over, registered at four family health centers located in the city center of Turkiye, which permitted the study to be conducted. The sample of the study consists of 356 older adults, calculated using the sampling method based on a known population (*N* = 4748, *p* = 0.50, q = 0.50, t = 1.96, d = 0.05). The sample included community-dwelling older adults who did not have any verbal communication disabilities (such as issues with perception, hearing, or speaking), were not dependent on others for daily living activities, and who consented to participate in the study. All older adults who visited the family health centers between January 2 and March, 2 2020 were invited to participate, reaching a total of 440 individuals. Of these, 8 were excluded due to hearing problems, and 32 declined participation, resulting in a final study sample of 400 community-dwelling older adults.

### Data collection instruments

Individual Evaluation Form, Attitudes to Ageing Questionnaire-Turkish Version (AAQ-TR), Geriatric Depression Scale and Templer Death Anxiety Scale were used to collect data.

- 1. *Individual Evaluation Form*: The Attitudes to Ageing Questionnaire was developed by the World Health Organization as a multi-center international project on behalf of Turkey. AAQ consists of 24 questions and 3 domains. These 3 domains include psychosocial loss (PL), physical change (PC) and psychosocial growth (PG). Each question is graded with a 5-point Likert-type scale with values between 1 and 5. The domains are assessed between 8 points at minimum and 40 points at maximum. Total score can also be calculated in addition to the domain scores. The higher the score, the better the perceived quality of life. After the score of psychosocial loss domain is reversed, the attitude score of the relevant domain also changes positively as the scale score increases. Cronbach alpha values of psychosocial loss, physical change and psychosocial growth are 0.75, 0.74 and 0.62, respectively [36]. In this study, the Cronbach alpha value was determined as 0.85 for the whole scale, and as 0.72 for the psychosocial loss, 0.75 for the physical change, and 0.61 for the psychosocial growth domains.
- 2.* Attitudes to Ageing Questionnaire Turkish Version(AAQ-TR)*: The Attitudes to Ageing Questionnaire was developed by the World Health Organization as a multi-center international project on behalf of Turkey. AAQ consists of 24 questions and 3 domains. These 3 domains include psychosocial loss (PL), physical change (PC) and psychosocial growth (PG). Each question is graded with a 5-point Likert-type scale with values between 1 and 5. The domains are assessed between 8 points at minimum and 40 points at maximum. Total score can also be calculated in addition to the domain scores. The higher the score, the better the perceived quality of life. After the score of psychosocial loss domain is reversed, the attitude score of the relevant domain also changes positively as the scale score increases. Cronbach alpha values of psychosocial loss, physical change and psychosocial growth are 0.75, 0.74 and 0.62, respectively [36]. In this study, the Cronbach alpha value was determined as 0.85 for the whole scale, and as 0.72 for the psychosocial loss, 0.75 for the physical change, and 0.61 for the psychosocial growth domains.
- 2. *Templer Death Anxiety Scale*: “Death Anxiety Scale” was developed by D.I. Templer [37] in 1970 in order to measure death anxiety. The validity and reliability studies of its Turkish version were carried out by Senol in 1989 and by Akca and Kose in 2008 [38]. It is a 15-item, true–false scale that measures an individual's anxiety and fears about her own death and risk of death. Each “yes” is graded as “1” and each “no” is graded as “0” for the first 9 items in the scale. For the other 6 items, “no” response was graded as “1” and “yes” was graded as “0”. Total score of the participant provides death anxiety score. The highest score that can be taken from the scale is 15. The assessment of death anxiety is made as “mild level” between 0–4, “moderate level” between 5–9, “high level” between 10–14 and “panic level” at 15 points. The coefficient of the original scale as determined by KR-20 formula was reported as 0.76 and product-moment correlation coefficient was reported as 0.83. In the study by Senol, internal consistency coefficient (Cronbach alpha) of the scale was reported as 0.72 and test–retest reliability coefficient was reported as 0.80. Based on the results of the study by Akca and Kose (2008), test–retest reliability of the scale was found to be 0.79 and the reliability coefficient calculated by KR-20 formula was found as 0.75; and these results support the relevance of the psychometric properties of the scale in the Turkish population [38]. In this study, Cronbach alpha value of the scale was determined as 0.77.
- 3. *Geriatric Depression Scale-15 (GDS-15)*:Geriatric Depression Scale, that was developed by Yesavage et al. (1982) consists of 30 questions [39]. Shiekh et al. (1986) created a short form of this scale including 15 questions that can be applied quickly and easily (GDS-15). GDS-15 includes 15 questions that query the patient’s emotional state [40]. The patients should answer the questions based on their feelings during the last week. The validity and reliability study of the Turkish version of GDS-15 was carried out by Durmaz et al. (2018). 5 questions of GDS-15 (1,5,7,11 and 13) were constructed as positive and 10 questions(2, 3, 4, 6, 8, 9, 10, 12, 14 and 15) were constructed as negative. In the evaluation of the scale, answers of “no” to positive questions and “yes” to negative questions were matched with 1 point. A score of 0–4 indicates no depressive symptoms, a score of 5–8 indicates mild depressive symptoms, a score of 9–11 indicates moderate depressive symptoms, and scores of 12 and above indicate severe depressive symptoms. The Turkish reliability and validity study of the scale was performed by Durmaz et al. (2018), and the Cronbach alpha value was found to be 0.920 [41]. In this study, the Cronbach Alpha value of the scale was determined as 0.82.

### Data collection

Data were collected between January 2 and March, 2 2020. After obtaining the approval of the ethics committee and the permission of the institution, commumity-dwelling older adults who applied to the family health centers where the study was conducted were informed about the purpose of the study. The data collection forms were applied to community-dwelling older adults, who met the study criteria and wanted to participate in the study, by the first researcher using the face-to-face interview technique.

### Data analysis

Data were analyzed with IBM SPSS Statistics for Windows version 25.0 (IBM Corp., Armonk, NY). The normality of the dataset was evaluated via Skewness–Kurtosis values, and it was found to be normally distributed. Descriptive statistics were presented via numbers, percentages, means, and standard deviation. The assumptions of the regression analysis were confirmed. Ageing attitude score and depression score as the predisposing factors of death anxiety were analyzed via the simple regression analysis. Significant independent variables suitable for the model were simultaneously included in the model using the "enter" method and multiple regression analysis was performed. Model summary was presented with R, R2, adjusted R2 and F, and analysis results were presented with standardized β and standard error. Results were reported as confidence intervals at 95% confidence intervals. The statistical significance level was accepted as *p* < 0.05.

## Results

Table 1 gives an overview of the characteristics of the community-dwelling older adults.

**Table 1:** The distribution of descriptive characteristics of community-dwelling older adults

Variables	n	%
**Age**		
65–74 years	268	67.0
75–84 years	119	29.7
85 years and older	13	3.3
**Gender**		
Female	178	44.5
Male	222	55.5
**Marital status**		
Married	236	59.0
Single	6	1.5
Widow (partner is dead)	151	37.7
Divorced	7	1.8
**Education status**		
Illiterate	71	17.7
Literate	86	21.5
Elementary school	112	28.0
Secondary school	42	10.5
High school	71	17.7
College/Faculty	17	4.3
Postgraduate	1	0.3
**Income status**		
Income less than expenses	246	61.5
Income equal to expenses	124	31.0
Income more than expenses	30	7.5
**People living together**		
Spouse	144	35.9
Alone	25	6.3
Spouse and children	93	23.3
Children	123	30.7
Close relative	15	3.8
**Chronic disease status**		
None	50	12.5
At least one	186	46.5
More than one	164	41.0
**Regular medication**		
None	31	7.8
At least one	124	31.0
More than one	245	61.2
**General health perception**		
Very good	8	2.0
Good	62	15.5
Moderate	164	41.0
Bad	141	35.2
Very bad	25	6.3
**Status of satisfaction from domestic environment**		
Satisfied	317	79.2
Dissatisfied	83	20.8

Table 2 gives an overview of the mean score of AAQ, GDS-15 and Templer Death Anxiety Scale. For the community-dwelling older adults, mean score of AAQ score was found as 65.16 (SD = 13.21). Mean scores of the domains were found to be 21.47 (SD = 5.61) for psychosocial loss, 18.62 (SD = 5.58) for physical change and 25.01 (SD = 4.50) for psychosocial growth. Mean score of the community dwelling older adults from GDS-15 was 7.25 (SD = 3.74). While no depressive findings were found in 28.0% (*n* = 112) of the older adults, 25.8% (*n* = 103) experienced mild, 35.8% (*n* = 143) experienced moderate level and 10.5% (*n* = 42) were found to show severe depressive symptoms. Mean score of the older adults from the Templer Death Anxiety Scale was determined as 8.45 (SD = 3.38). Death anxiety was found to be experienced at a mild level in 13.8% (*n* = 55), at a moderate level in 44.3% (*n* = 177), at a high level in 41.8% (*n* = 167) and at a panic level in 0.3% (*n* = 1) of the older adults.

**Table 2:** Distribution of the scores of AAQ, GDS-15 and Templer Death Anxiety Scale among community-dwelling older adults

Scale	Number of items	Mean	StandardDeviation	Min	Max
**Attitude to Ageing Questionnaire**					
Total	24	65.16	13.21	33	105
Psychosocial Loss	8	21.47	5.61	8	37
Physical Change	8	18.62	5.58	8	34
Psychosocial Growth	8	25.01	4.50	10	37
**Geriatric Depression Scale-15**					
Total	15	7.25	3.74	0	15
No depression	28.0% (*n* = 112)				
Mild depressive symptoms	25.8% (*n* = 103)				
Moderate level depressive symptoms	35.8% (*n* = 143)				
Severe depressive symptoms	10.5% (*n* = 42)				
**Templer Death Anxiety Scale**					
Total	15	8.45	3.38	0	15
Mild level	13.8% (*n* = 55)				
Moderate level	44.3% (*n* = 177)				
High level	41.8% (*n* = 167)				
Panic level	0.3% (*n* = 1)				

Simple and multivariate linear regression analysis was performed to evaluate the predictors of death anxiety in the older adults (Table 3). As a result of this analysis, it was found that a significant regression model, F (2, 397) = 93.427, *p* < 0.001 and 32% of the variance in the dependent variable (R2 adjusted = 0.317) were explained by the independent variables. According to the analysis results, ageing attitude score (β = -0.154, *p* = 0.001) and depression score (β = 0.490, *p* < 0.001) were found to affect death anxiety of the older adults at a significant level. Based on this finding, the increase in the negative attitudes to ageing and the level of depression are suggested to increase death anxiety of the older adults.

**Table 3:** Attitude to ageing and depression as the predictors of death anxiety

Variables	Simple model					Multiple model				
	(95% CI for B)					(95% CI for B)				
	B	Lower	Upper	β	p	B	Lower	Upper	β	p
**Attitude to Ageing Questionnaire**	-0.086	-0.110	-0.063	-0.337	** < 0.001**	-0.039	-0.062	-0.017	-0.154	**0.001**
**Geriatric Depression Scale-15**	0.495	0.421	0.570	0.547	** < 0.001**	0.443	0.364	0.522	0.490	** < 0.001**
**R**						0.566				
**R2**						0.320				
**R2 Adjusted**						0.317				
**F and p value**						93.427, < **0.001**				

## Discussion

To the best of our knowledge, this study was the first one in which attitudes to ageing and depression are evaluated together as predictors of death anxiety in the community-dwelling older adults. The main findings of this study are as follows:

In the present study, mean scores of the subscales of attitude to ageing including psychosocial loss, physical change, and psychosocial growth were found to be 21.47, 18.62 and 25.01, respectively. Similarly, in a study conducted in Turkey, Top et al. reported mean scores of psychosocial loss, physical change and psychosocial growth as 26.5, 24.48 and 26.00, respectively [28]. In another study by Korkmaz et al., it was found that mean scores of psychosocial loss, physical change and psychosocial growth were 28.68, 24.47 and29.77, respectively. The results of this current study seem to be similar to the results of other two studies; but mean score of physical change domain was indicated to be smaller. Unlike Turkish sample studies, previous studies have reported higher mean scoresin physical change domain [42, 43]. According to the previous studies, it can be suggested that the current study results have the potential to be generalized to a broader group of Turkish older adults.

Depression is a common health problem faced by older adults. According to the United States epidemiological survey, geriatric depression affects 8% to 15% of older adults. Moreover, the prevalence of geriatric depression was reported as 12% to 34% among older people in Asian countries [44]. Furthermore, in southern Taiwan, the prevalence of geriatric depression was found to be between 12.9% and 21.7% among older adults [45]. Similarly, majority of older adults were found to have mild, moderate and severe depressive symptoms in this current study. Despite the differences in the sample, it might be said that depression affects a broader group of older adults.

In this present study, death anxiety scale score was found as 8.45 + 3.38, and the majority of the older adults were found to have death anxiety at a moderate or higher level. Similarly, previous studies have shown that older adults have death anxiety at a moderate and higher level [46–49]. Based on these results, it can be suggested that older adults experience death anxiety in different countries and different cultures.

According to the results of regression analysis, depression was determined as the most important predicting factor of death anxiety in this study. In the literature, it has been argued that individuals with depression tend to experience death anxiety more because they experience more negative emotions [50]. Moreover, it has been stated that negative thoughts of the individuals cause hopelessness and cognitive distortionsas the depressive symptoms intensify, leading to an increase in the level of death anxiety [19, 20, 48]. The findings of this present study are in line with the results of previous studies. Therefore, it can be said that depressive symptoms increase the level of death anxiety among older adults. This study also has shown that attitude to ageing is a predicting factor of the death anxiety. Similarly, Mohommadpour et al. found that death anxiety score was predictable according to the all dimensions of perception of ageing in the older adults. It was stated that accepting the natural ageing process and the positive or negative situations that this process bring can reduce death anxiety [51]. Similarly, Kahraman and Erkent (2022) found that the attitude to ageing and older age had a significant mediating role in the effect of the meaning and purpose of life on death anxiety. They have stated that negative attitudes to ageing and death anxiety increase due to the fact that people think about old ageas closer to death although death can be seen in all age groups. Moreover, they have indicated that the death of spouses, friends and relatives in old age can make elder individuals to think about old age more negatively and experience death anxiety [52]. Although the research on attitudes to ageing and death anxiety is limited, it can be said that negative attitudes to ageing cause an increase in death anxiety based on the results of these three studies.

Moreover, the adjusted R^2^ value of 0.317 indicates that there are other factors, not included in the current model, which may influence death anxiety. Future studies could explore additional factors that might contribute to a more comprehensive understanding of death anxiety in older adults. These factors may include social support, physical health conditions, cultural beliefs, religious practices, personality traits, life satisfaction, and previous experiences with loss. By including these factors in future studies, a more nuanced understanding of death anxiety could be developed.

## Limitations

The limitations of this study should also be considered. Specifically, the cross-sectional design limits the ability to draw causal inferences. Additionally, the findings may not be generalizable, as the study was conducted in a single city in Turkiye with a specific sample of older adults, which may restrict the applicability of the results to other populations or cultural contexts. Since participants’ perceptions of their attitudes toward ageing, depressive symptoms, and death anxiety were based on self-reported data, they may not fully capture the actual situation.

## Conclusion

In line with the results of this study, it can be concluded that majority of community-dwelling older adults have depressive symptoms and death anxiety; and the attitudes to ageing and depression can predict death anxiety among community-dwelling older adults. The study also showed the importance of attitudes to ageing and depressive symptoms in alleviating death anxiety among older adults. This result highlights that interventions should be planned in order to reduce death anxiety among community-dwelling older adults by taking the attitude to ageing and the symptoms of depression into account.

### Implications for practice

The results of the present study have important implications for nursing practice and future research. Nurses are the primary healthcare workers for the early detection of depression and death anxiety among older adults [48]. Thus, they should regularly assess older adults for their attitude to ageing, depression, and anxiety coping. Moreover, nurses can motivate them through regular counseling and screening. There is an increasing focus on health promotion for ageing population, and nurses have a crucial role in this regard. Nurses, who work in the primary care office, should assess and monitor the symptoms of older adults, provide education to community-dwelling older adults and their families, schedule activities, and communicate with all care providers [29]. Thus, nurses will not only be able to promote the health of older adults, but also contribute to a better quality of life.

On the other hand, there is a need for a social policy about attitudes to ageing and older adults. Governments and policymakers should strive to build community-focused intervention programs which include promoting age-friendly and community-based health care programs for older people and using community resources that are directed toward the older population. Moreover, policies that challenge ageing stereotypes should be developed not only for older adults but also for people of all ages in order to promote more positive attitudes to ageing. This will help to improve older adults’ positive attitudes to ageing and decrease their depression levels and death anxiety.

Further studies may use qualitative methods to investigate older people’s attitudes and life experiences about ageing in depth so that a wider understanding of the ageing process can be accessed from older adults’ own perspectives.

## Data Availability

The datasets generated during and/or analysed during the current study are available from the corresponding author on reasonable reques.
